# Identification of Main Influencers of Surgical Efficiency and Variability Using Task-Level Objective Metrics: A Five-Year Robotic Sleeve Gastrectomy Case Series

**DOI:** 10.3389/fsurg.2022.756522

**Published:** 2022-05-02

**Authors:** Mark R. Tousignant, Xi Liu, Marzieh Ershad Langroodi, Anthony M. Jarc

**Affiliations:** ^1^Medical Safety and Innovation, Intuitive Surgical Inc., Sunnyvale, CA, United States; ^2^Applied Research, Intuitive Surgical Inc., Peachtree Corners, GA, United States

**Keywords:** robotic-assisted surgery, sleeve gastrectomy, objective performance indicators, surgical task, workflow analysis, video analytics

## Abstract

**Objective:**

Surgical efficiency and variability are critical contributors to optimal outcomes, patient experience, care team experience, and total cost to treat per disease episode. Opportunities remain to develop scalable, objective methods to quantify surgical behaviors that maximize efficiency and reduce variability. Such objective measures can then be used to provide surgeons with timely and user-specific feedbacks to monitor performances and facilitate training and learning. In this study, we used objective task-level analysis to identify dominant contributors toward surgical efficiency and variability across the procedural steps of robotic-assisted sleeve gastrectomy (RSG) over a five-year period for a single surgeon. These results enable actionable insights that can both complement those from population level analyses and be tailored to an individual surgeon's practice and experience.

**Methods:**

Intraoperative video recordings of 77 RSG procedures performed by a single surgeon from 2015 to 2019 were reviewed and segmented into surgical tasks. Surgeon-initiated events when controlling the robotic-assisted surgical system were used to compute objective metrics. A series of multi-staged regression analysis were used to determine: if any specific tasks or patient body mass index (BMI) statistically impacted procedure duration; which objective metrics impacted critical task efficiency; and which task(s) statistically contributed to procedure variability.

**Results:**

Stomach dissection was found to be the most significant contributor to procedure duration (β = 0.344, *p*< 0.001; *R* = 0.81, *p*< 0.001) followed by surgical inactivity and stomach stapling. Patient BMI was not found to be statistically significantly correlated with procedure duration (*R* = −0.01, *p* = 0.90). Energy activation rate, a robotic system event-based metric, was identified as a dominant feature in predicting stomach dissection duration and differentiating earlier and later case groups. Reduction of procedure variability was observed between earlier (2015-2016) and later (2017-2019) groups (IQR = 14.20 min vs. 6.79 min). Stomach dissection was found to contribute most to procedure variability (β = 0.74, *p* < 0.001).

**Conclusions:**

A surgical task-based objective analysis was used to identify major contributors to surgical efficiency and variability. We believe this data-driven method will enable clinical teams to quantify surgeon-specific performance and identify actionable opportunities focused on the dominant surgical tasks impacting overall procedure efficiency and consistency.

## Introduction

Surgical efficiency and variability are critical contributors to optimal outcomes, patient and care team experience, and total cost to treat per disease episode (1–3). However, it is often unclear to clinical teams how to objectively quantify their own surgical efficiency and variability. Further, population-level analyses alone are not always able to deliver actionable insights to an individual surgeon due to unique aspects during practice. Therefore, objective methods to characterize surgical workflow and identify actionable areas for improvement with tailored feedback for each surgeon still need to be developed and made widely available.

Although multiple factors influence outcomes and efficiencies, many studies focus on how surgery is performed by describing subjectively initial case series or critical aspects within the procedure. Few studies use objective methods to identify which surgical activities and how surgeon performance affect overall procedure efficiency or surgical outcomes throughout a surgeon's learning curve (4–8). These studies are largely agnostic to the underlying surgical activities by using global subjective rating scales like Global Evaluative Assessment of Robotic Skills (GEARS) (9) or Objective Structured Assessment of Technical Skills (OSATS) (10). Further, although some studies describe the tasks within a surgery (11–13), there is room for improvement through the establishment of quantitative methods to provide actionable objective measures. Finally, task-based objective performance indicators (OPIs) other than total operative time are often neglected despite offering the potential for improved and focused feedback (14–17). There exists an opportunity to develop more objective methods that can scale for broad use (18–20) given a limited number of studies use subjective methods to estimate the impact of a surgeon's technical skills on patient outcomes (21–25). Additionally, these objective methods need to be able to be applied to an individual surgeon, within institutions, or across institutions.

The purpose of this study was to demonstrate a novel, data-driven method that retrospectively identifies dominant factors that influenced a surgeon's performance efficiency and variability over five years when performing robotic-assisted sleeve gastrectomy (RSG) procedures. Specifically, we (1) identified the dominant factors of surgical efficiency and variability within RSG by focusing on surgical tasks, (2) examined the influence of body mass index (BMI), an important patient factor within bariatric surgery (26), on efficiency, and (3) identified OPIs with greatest impact on efficiency of the identified critical step. The data-driven methods developed in this study might also be further generalized for clinical teams, residents during training and educators to quantify performance and identify actionable and scalable changes.

## Materials and Methods

### Study Design

**Figure 1:**
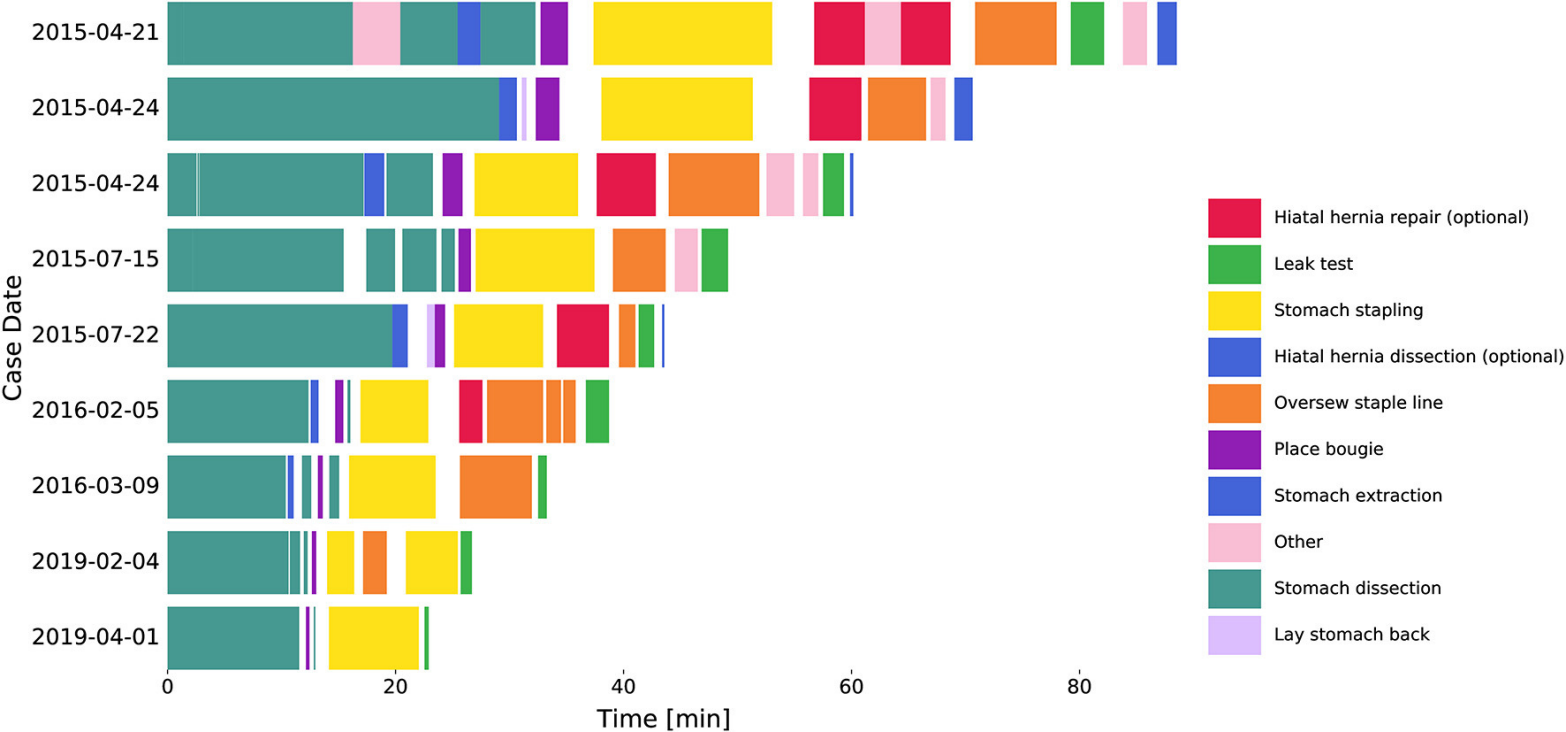
Procedure workflow changes over years. Segmented tasks from 9 example cases. Hiatal hernia dissection and repair were excluded from further analysis. Surgical inactivity time was denoted as the gaps between tasks.

Seventy-seven RSG procedures performed by a single surgeon from April 21st, 2015 to June 3rd, 2019 were retrospectively reviewed. All procedures were performed using the da Vinci Xi surgical system (Intuitive Surgical Inc., Sunnyvale, CA, USA). Nine surgical tasks were defined that constitute the major steps needed to complete a sleeve gastrectomy (Figure 1). The tasks included stomach dissection, hiatal hernia dissection (optional), lay stomach back, place bougie, stomach stapling, hiatal hernia repair (optional), oversew staple line, leak test and stomach extraction. Any additional surgical activities were defined as “other” and idle time between tasks were defined as “surgical inactivity.”

Detailed criteria for task start and stop times was also defined to minimize annotation variability. For example, the start time of stomach dissection was defined as the time when a dissection tool engages with tissue to initiate dissection along the greater curve of the stomach. The start and stop times for each task in each video were then annotated by three professionally trained annotation technicians. An expert surgeon reviewed samples of these annotations to ensure quality. Note that hiatal hernia dissection and repair were optional tasks in RSG procedures and thus excluded from procedure time and subsequent analysis.

To observe and compare the changes in task completion time, we grouped the surgical videos into earlier and later case groups. Specifically, earlier cases included 39 videos from the years 2015 through 2016, and the later cases included 38 videos from the years 2017 through 2019. Note that the earlier cases were a subset from the first 50 cases of the surgeon and the later cases were a subset from the latest 80 cases of the same surgeon.

### Procedure Efficiency and Variability

Overall procedure duration was considered as a measure of efficiency, and interquartile range (IQR) of consecutive case durations was used as a measure of variability. To further study the efficiency of the identified task(s), surgeon behavior was characterized by OPIs derived from three major surgical robotic system events: camera movement, energy activation and arm swap. The start and stop timestamps for each event were used to calculate event-based OPIs, including rates of occurrences, and median durations of all occurrences. Identifying the OPIs that contribute to overall improvement of the task assists with identifying the skills that need to be focused on during training to improve efficiency.

### Statistical Analysis

We used a three-staged regression analysis to identify main contributor(s) to procedure efficiency: (1) Spearman rank-order correlation test between each independent variable and procedure duration; (2) multivariable regression analysis; (3) recursive feature elimination (RFE) (27). The variables considered in efficiency analysis were task durations and patient BMI. Specifically, the correlation matrix of all independent variables was first checked to ensure no multicollinearity in the data. Task duration, procedure duration, and BMI were then normalized by corresponding median values from the first 5 cases to capture a baseline of surgeon behavior and patient factors. Next, β coefficients from a multivariable linear regression analysis were compared to identify variable(s) with the highest impact on procedure efficiency. Finally, RFE was used to rank the independent variables. This analysis leads to identifying the critical task that can be focused on for further analysis. Confounding effect of BMI on the critical task in association with procedure efficiency was also examined.

To characterize the impact of surgeon behavior on efficiency, we computed event-based OPIs for the identified critical task and investigated the association between OPIs and task duration using the three-staged regression analysis. We also evaluated the ability of these OPIs in differentiating between earlier and later case groups using logistic regression. RFE and LASSO (28, 29) feature selection methods were again used to rank the OPIs.

Finally, we examined association between procedure and task duration variability across all procedures. IQRs of procedure and task durations were computed by applying a sliding window for every five consecutive procedures with a stride of one procedure in earlier and later groups, respectively. Task(s) that contributed most to overall procedure variability was then identified using the same three-staged analysis. Furthermore, logistic regression with RFE was used to identify tasks with most variability between earlier and later groups. *p* < 0.05 was considered statistically significant in all of our statistical analysis. Statistical analysis was performed using Python's statistical functions (Python 3.7.9; SciPy v1.5.2; scikit-learn 0.23.2).

## Results

### Procedure Characteristics

**Figure 2:**
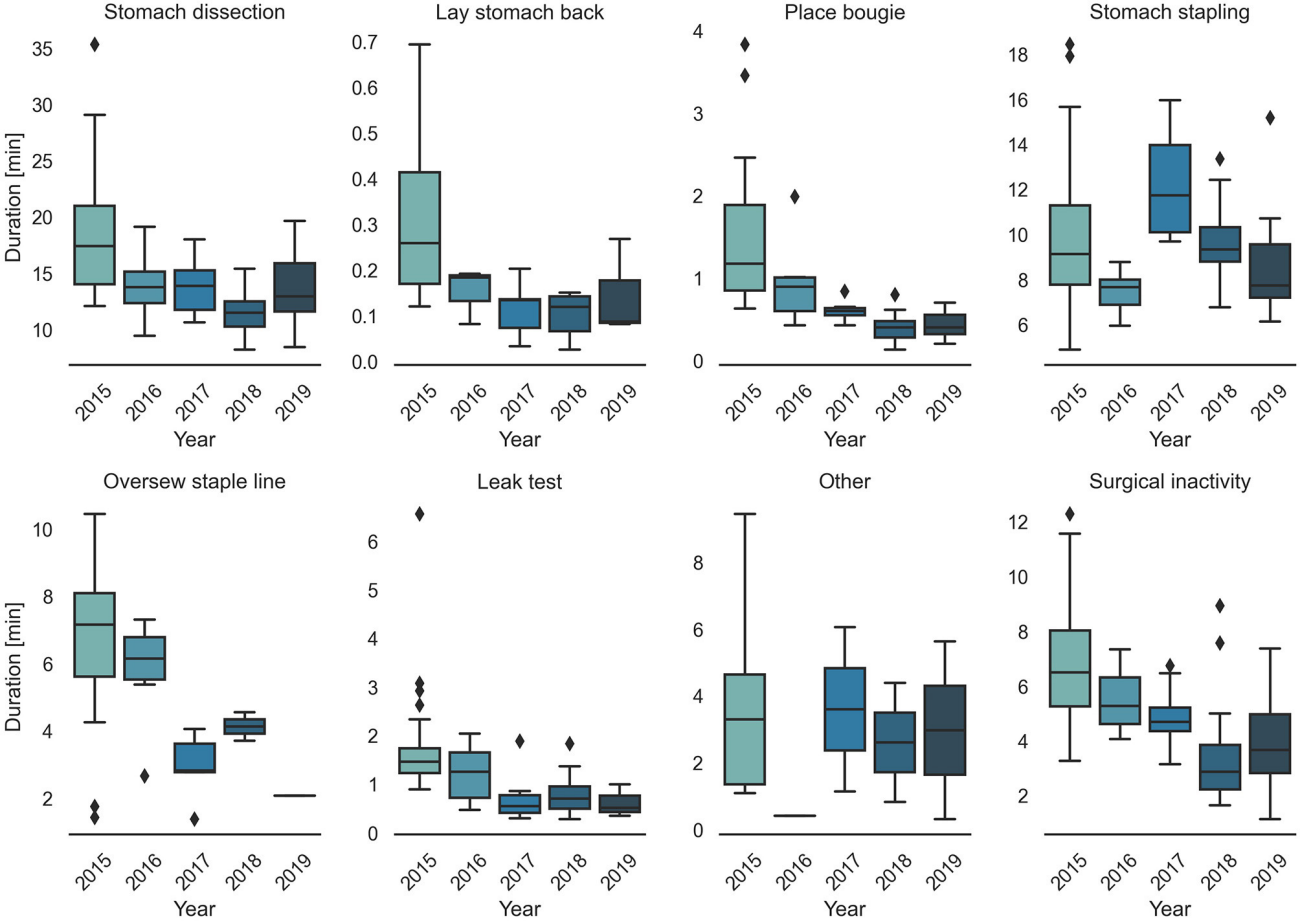
Trend in task duration change of all 77 cases over five years.

Surgical task annotation results of nine example cases ordered chronologically were shown in Figure 1. Each row corresponded to one case and each color bar corresponded to an annotated task in the case. Reductions in procedure duration and task duration and variability can also be observed as the surgeon progressed over years (Figures 1, 2).

**Figure 3:**
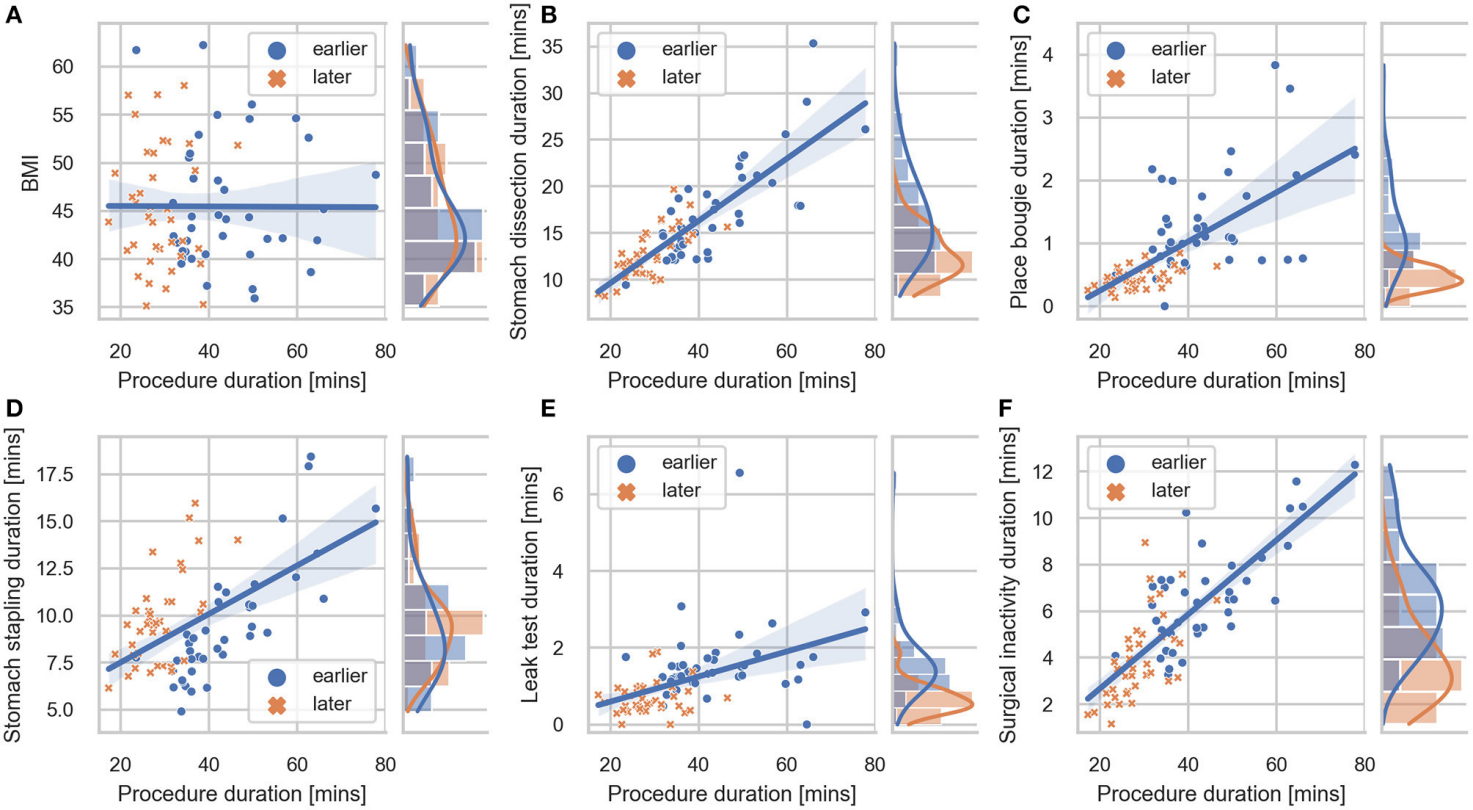
Correlation plot between BMI, task durations and procedure duration. Regression lines are included for each sub-comparison. The 95% confidence intervals were shown as the translucent bands around the regression line. Distributions of **(A)** BMI, **(B)** stomach dissection, **(C)** place bougie, **(D)** stomach stapling, **(E)** leak test, **(F)** surgical inactivity with regard to procedure durations are included for earlier and later case groups, respectively.

Detailed characteristics of the case series, including the number of occurrences, median value and IQR of different case groups were provided in Table 1. Among the seven surgical tasks, five tasks were identified as frequent tasks across the case series: stomach dissection, place bougie, stomach stapling, oversew staple line and leak test, with occurrences of oversew staple line decreased [earlier 36 (92.3%) vs. later 8 (21.1%)]. When comparing earlier and later case groups at procedure level, median procedure duration and IQR decreased (earlier 41.89 min, IQR = 14.2 min vs. later 27.73 min, IQR = 6.79 min). Similarly, median duration of all frequent tasks decreased except for stomach stapling, and IQRs of all five frequent tasks decreased. The decreases in both median durations and IQRs indicates procedure efficiency improvement and variability reduction between the earlier and later groups. There is no obvious change in patient BMI characteristics (earlier 44.14, IQR = 8.85 vs. later 44.25, IQR = 9.62). Distribution of BMI and procedure time can be found in Figure 3.

**Table 1:** Statistics of surgical tasks, procedure duration and BMI.

	**No. (%)**		**Median Value**^**a**^ **(IQR)**			
	**Earlier (*n* = 39)**	**Later (*n* = 38)**	**First 5 cases^**b**^**	**Earlier**	**Later**	**All cases**
Stomach dissection	39 (100%)	38 (100%)	21.18 (5.75)	16.45 (6.57)	12.23 (4.13)	14.19 (5.29)
Lay stomach back	18 (46.2%)	14 (36.8%)	0.42 (0.00)	0.21 (0.22)	0.12 (0.07)	0.16 (0.14)
Place bougie	38 (97.4%)	38 (100%)	1.75 (1.35)	1.09 (0.98)	0.43 (0.24)	0.68 (0.66)
Stomach stapling	39 (100%)	38 (100%)	15.15 (2.39)	8.79 (3.16)	9.52 (2.79)	9.15 (3.05)
Oversew staple line	36 (92.3%)	8 (21.1%)	7.96 (1.79)	6.90 (2.61)	3.23 (1.19)	6.25 (3.41)
Leak test	38 (97.4%)	36 (94.7%)	2.24 (1.03)	1.42 (0.56)	0.60 (0.43)	1.07 (0.92)
Stomach extraction	10 (25.6%)	0 (0%)	0.55 (1.11)	0.42 (0.70)	0.00 (0.00)	0.42 (0.70)
Other	12 (30.8%)	6 (15.8%)	4.84 (3.61)	2.66 (3.09)	2.77 (4.42)	2.66 (3.79)
Surgical inactivity	39 (100%)	38 (100%)	8.81 (3.28)	6.45 (2.23)	3.52 (2.29)	5.03 (3.29)
Total procedure	39	38	62.54 (7.81)	41.89 (14.20)	27.73 (6.79)	34.37 (14.57)
BMI	39	38	42.17 (6.65)	44.14 (8.85)	44.25 (9.62)	44.14 (9.71)

a *For task and procedure, median durations (IQR) in minutes from all non-zero occurrences were reported. For BMI (calculated as weight in kilograms divided by height in meters squared), median value (IQR) was reported*.

b *The values from the first 5 cases were used as baseline for normalization during the analysis*.

### Efficiency Analysis

#### Critical Task Identification

In the first-stage analysis, none of the independent variables were found to be highly correlated with each other (Spearman rank-order correlation coefficients R ranging from −0.24 to 0.66) (detailed correlation matrix is visualized in Supplementary Figure 1). Correlation coefficients between each variable and procedure duration were summarized in Table 2 and visualized in Figure 3. Among all variables, stomach dissection was found to be most significantly correlated with procedure duration (*R* = 0.81, *p* < 0.001). BMI was not found to be statistically significantly correlated with procedure duration (R = −0.01, *p* = 0.90).

**Table 2:** Regression models examining procedure duration change by surgical task duration and BMI change.

	**Spearman correlation**		**Multivariable linear regression**								
	**All cases**		**Earlier**			**Later**			**All cases**		
	**R**	***p*** **value**	**β Coefficient (95% CI)**	***p*** **value**	**RFE rank**	**β Coefficient (95% CI)**	***p*** **value**	**RFE rank**	**β Coefficient (95% CI)**	***p*** **value**	**RFE rank**
Stomach dissection	0.81	**<0.001**	0.343 (0.324 to 0.362)	**<0.001**	2	0.337 (0.334 to 0.340)	**<0.001**	1	0.344 (0.333 to 0.355)	**<0.001**	1
Place bougie	0.74	**<0.001**	0.031 (0.022 to 0.041)	**<0.001**	6	0.027 (0.023 to 0.032)	**<0.001**	7	0.031 (0.025 to 0.037)	**<0.001**	6
Stomach stapling	0.39	**<0.001**	0.244 (0.216 to 0.272)	**<0.001**	1	0.246 (0.244 to 0.248)	**<0.001**	2	0.246 (0.235 to 0.258)	**<0.001**	3
Oversew staple line	0.78	**<0.001**	0.129 (0.117 to 0.141)	**<0.001**	4	0.128 (0.126 to 0.131)	**<0.001**	4	0.129 (0.123 to 0.135)	**<0.001**	4
Leak test	0.55	**<0.001**	0.028 (0.021 to 0.035)	**<0.001**	7	0.030 (0.028 to 0.032)	**0.035**	6	0.029 (0.024 to 0.033)	**<0.001**	7
Other	0.45	**<0.001**	0.059 (0.050 to 0.069)	**<0.001**	5	0.061 (0.060 to 0.062)	**<0.001**	5	0.061 (0.056 to 0.065)	**<0.001**	5
Surgical inactivity	0.74	**<0.001**	0.158 (0.135 to 0.182)	**<0.001**	3	0.140 (0.138 to 0.143)	**<0.001**	3	0.149 (0.138 to 0.159)	**<0.001**	2
BMI	−0.01	0.90	0.004 (−0.025 to 0.033)	0.79	8	0.002 (−0.001 to 0.005)	0.17	8	0.001 (−0.012 to 0.014)	0.88	8

*Bold values are corresponding to statistically significant p-values*.

In the subsequent multivariable regression analysis, all independent variables were normalized by corresponding median durations of the first 5 cases from the surgeon (Table 1) to ensure fair comparison. The β coefficients of each variable were compared among earlier, later and all cases (Table 2). In the earlier group, a unit increase in stomach dissection duration relative to the median duration from the first 5 cases (i.e., increases by 21.18 min) was associated with a 34.3% increase (β = 0.343, 95% CI 0.324 to 0.362, *p* < 0.001) in baseline procedure duration (i.e., a 34.3% increase from 62.54 min). Compared with all other variables, stomach dissection was found to be associated with the largest β coefficient. Similarly, when considering later cases and all cases, stomach dissection was again associated with the largest β coefficients (Table 2). Surgical inactivity also contributed to procedure duration increase in both earlier and later groups (earlier β = 0.158, 95% CI 0.135 to 0.182, *p* < 0.001 vs. later β = 0.140, 95% CI 0.138 to 0.143, *p* < 0.001). In contrast, BMI was not found to be statistically significant in association with procedure duration in all cases (β = 0.001, 95% CI −0.012 to 0.014, *p* = 0.88; *R* = −0.01, *p* = 0.90) and neither in earlier or later groups.

Finally, RFE with linear regression was used to recursively eliminate and rank these eight features in predicting procedure duration change. Stomach dissection, stomach stapling and surgical inactivity were consistently ranked the top three most important features (Table 2). Patient BMI was consistently ranked the lowest across all groups.

Overall, stomach dissection was found to be the major critical task and main contributor to procedure efficiency considering all three stages of analysis. To further examine confounding effect of BMI on stomach dissection, β coefficient of dissection from a univariate linear regression (β = 0.703, 95% CI 0.595 to 0.810, *p* < 0.001) was compared to the coefficient from a multivariable regression model after adding BMI (β = 0.711, 95% CI 0.604 to 0.819, *p* < 0.001). The results indicate a 1.14% increase in the coefficient thus showing no confounding effect of BMI on stomach dissection task.

#### Event-Based Objective Performance Indicator

Five event-based OPIs were computed from surgical system events that occurred during stomach dissection. To investigate the association between OPIs and the critical task (i.e. stomach dissection) efficiency, the same three-staged regression analysis was performed. The absolute values of the correlation coefficients R between each pair of OPIs were in the range of (0.01, 0.44) ensuring no multicollinearity (Supplementary Figure 2). Energy activation rate, median duration of camera movement and camera movement rate were found to be statistically correlated with stomach dissection duration (Table 3). In the subsequent multivariable regression analysis, all variables were normalized to the median of the first 5 cases. Among all variables, energy activation rate was found to be statistically significantly associated with task duration (β =-2.40, 95% CI −3.90 to −0.91, *p* = 0.002). Finally, RFE was performed along with the linear regression to rank OPIs in association with stomach dissection duration. The rankings indicate that median duration of camera movement and energy activation rate were the two most influential OPIs on task efficiency. Overall, energy activation rate was found to be a consistent indicator of task efficiency considering all three-staged analyses.

**Table 3:** Regression models examining stomach dissection duration change by event OPIs.

**OPIs**	**Spearman correlation**		**Multivariable linear regression**			**Logistic regression**	
	**R**	***p*** **value**	**β Coefficient (95% CI)**	***p*** **value**	**RFE rank**	**RFE rank**	**LASSO coefficient**
Energy activation rate, counts per minute	−0.65	**<0.001**	−2.40 (−3.90 to −0.91)	**0.002**	2	1	4.21
Median of energy duration, seconds	0.15	0.26	0.58 (−0.36 to 1.53)	0.221	5	4	0
Camera movement rate, counts per minute	−0.29	**0.02**	−1.1 (−2.83 to 0.64)	0.211	4	3	0.32
Median duration of camera movement, seconds	0.50	**<0.001**	2.08 (−2.28 to 6.45)	0.341	1	2	0
Arm swap rate, counts per minute	−0.15	0.27	1.36 (−2.30 to 5.02)	0.459	3	5	0

*Bold values are corresponding to statistically significant p-values*.

**Figure 4:**
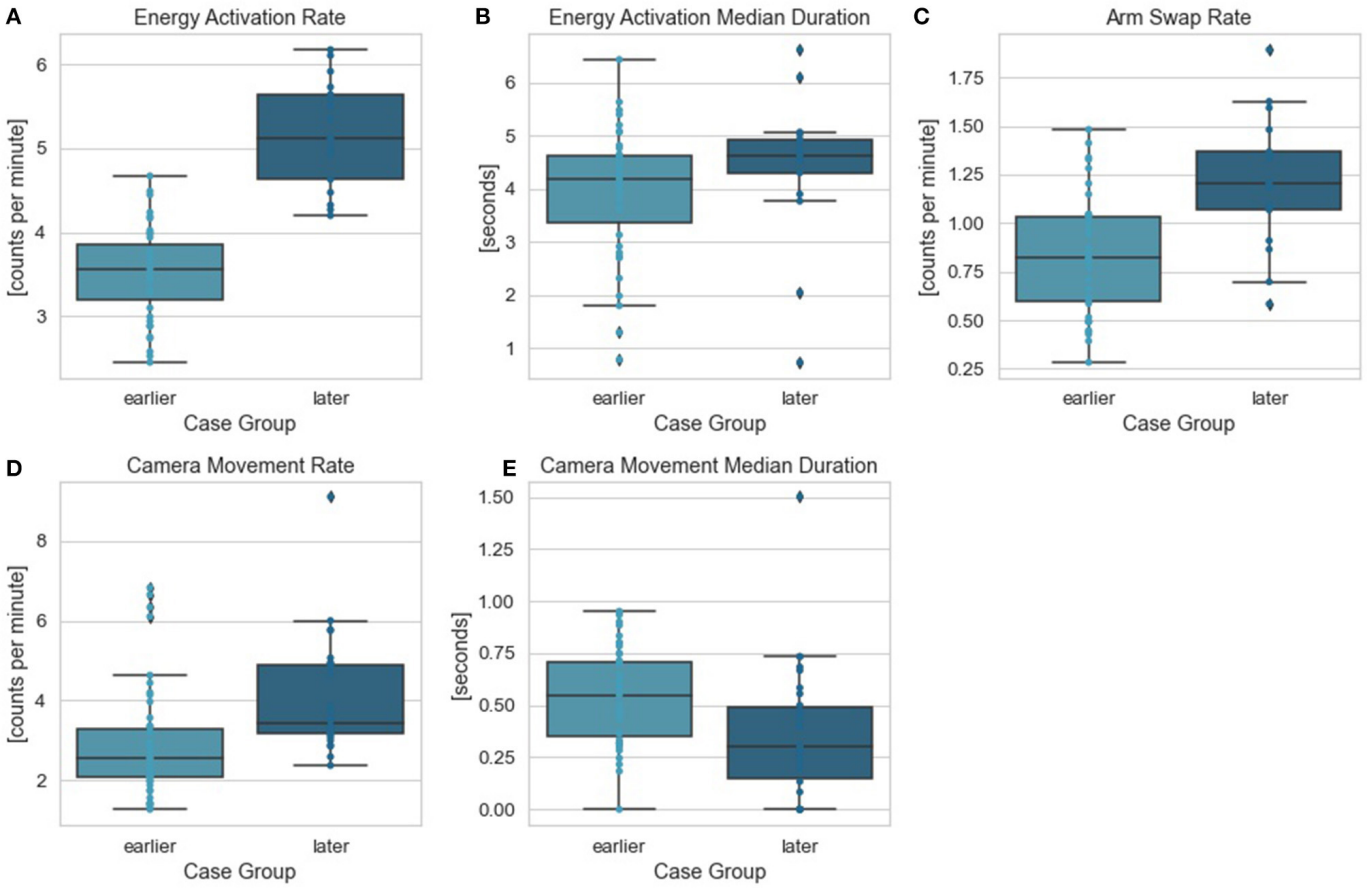
Stomach dissection OPIs between earlier and later case groups. Comparisons between earlier and later cases groups were provided for OPIs: **(A)** energy activation rate, **(B)** energy activation median duration, **(C)** arm swap rate, **(D)** camera movement rate, **(E)** camera movement median duration.

To further investigate surgeon's behavior change throughout the longitudinal dataset, we identified the OPIs that can best differentiate surgeon's performance in the critical task between earlier and later case groups. Two feature selection methods (LASSO and RFE) with logistic regression were used. Energy activation rate was again selected as the top feature by both methods. All features along with their ranks from RFE feature selection and coefficients from LASSO feature selection were summarized in Table 3. The comparisons of all OPIs for the earlier and later case groups were shown in Figure 4.

### Variability Analysis

We observed decreases in IQRs of procedure and task durations between earlier and later groups (Table 1). To further investigate the association between task and procedure duration variability, IQRs of task and procedure durations were computed for every five consecutive cases in earlier and later groups. The IQRs were then combined to analyze variability among all cases. Five tasks were selected as independent variables to ensure equal occurrences between earlier and later groups. To compare different tasks, all IQRs were normalized by the values from the first five cases (Table 1). None of the independent variables were found to be highly correlated (coefficients ranging from −0.18 to 0.33) (see Supplementary Figure 3).

When considering all cases, stomach dissection variability was found to contribute most to procedure variability with high consistency according to our three-staged analysis (Table 4). Specifically, a unit increase in stomach dissection IQR from 5 consecutive cases compared to the baseline IQR (Table 1) was associated with a 74% increase (β = 0.74, 95% CI 0.31 to 1.17, *p* = 0.001) in procedure duration IQR (i.e. a 74% increase from the baseline IQR = 7.81 min). Finally, stomach dissection and surgical inactivity were among the top three features in predicting procedure variability ranked by RFE.

**Table 4:** Regression models examining procedure variability by surgical task variability.

	**Spearman correlation**		**Multivariable linear regression**			**Logistic regression**
	**R**	***p*** **value**	**β Coefficient (95% CI)**	***p*** **value**	**RFE rank**	**RFE rank**
Stomach dissection	0.45	**<0.001**	0.74 (0.31 to 1.17)	**0.001**	1	4
Place bougie	0.26	**0.03**	0.07 (0.43 to 0.58)	0.78	5	1
Stomach stapling	0.30	**0.01**	0.25 (0.04 to 0.46)	**0.02**	4	5
Leak test	−0.16	0.20	−0.59 (−1.38 to 0.19)	0.14	2	2
Surgical inactivity	0.26	**0.03**	0.52 (0.09 to 0.96)	**0.02**	3	3

*Bold values are corresponding to statistically significant p-values*.

To identify tasks with most variability between earlier and later groups, we used RFE with logistic regression. These results showed that place bougie, leak test and surgical inactivity contributed most to variability differences between the two groups (Table 4).

## Discussion

We believe this study outlines a novel method to identify the dominant influencers to overall procedure efficiency and variability within RSG through surgical task decomposition and task-based OPIs. The multi-staged regression analysis can help to identify dominant factors that influence surgery through quantitative measures, which is critically important to delivering actionable and focused surgeon-specific feedback but may also be generalized to enable objective and scalable insights across institutions. These objective and scalable feedbacks could also be especially helpful for surgeons during training.

In order to gain a deeper insight into RSG, the procedures were segmented into nine distinct surgical tasks based upon clinical relevance, consistency across the case series, and the ability to establish clear definition of start and stop times. Moreover, the nine surgical steps were defined in such a way to accommodate for minor technique changes over the case series (i.e., hiatal hernia dissection and repair and oversew the staple line were not present in all procedures). Stomach dissection and gastric sleeve stapling were two critical tasks within RSG. Additional surgical activities beyond the nine distinct tasks were classified as other or surgical inactivity. The surgical task segmentation is a foundational component that enables the ability to perform focused and granular analysis than conventional learning curve analysis (8, 30, 31) for this RSG case series.

The multi-staged regression analysis was first used to analyze the case series to determine the critical surgical task impacting overall efficiency and variability. As one might expect, overall variability decreased as overall efficiency increased. The critical task the correlates highest with the total procedure efficiency and variability for this single surgeon RSG case series was identified as stomach dissection (Tables 2, 4). Stomach dissection requires a combination of clinical judgment, such as identification of the gastromesenteric ligament, pylorus, and short gastric vessels, as well as technical skill, such as energy use, retraction, dissection, and camera control. Education around clinical knowledge and technique and associated technical skills for this step offer an opportunity for focused gains on efficiency.

Surgical inactivity was another important factor impacting overall efficiency. Efforts to reduce periods of inactivity can be pursued by both the surgeon and OR team by reducing interruptions and training around the equipment and technique required to complete the procedure. Development of repeatable techniques, surgical approach, proficiency, and coordination by both the operating surgeon and OR team are essential to ensure consistency and predictability.

Notably, patient BMI consistently ranked the least dominant feature to impact total operative time. One possible explanation may be due to the fact that these cases were performed robotically, which may eliminate the ergonomic challenges of operating on high-BMI patients seen in conventional laparoscopy, a compelling result within robotic-assisted bariatric surgery. This finding is consistent with those reported in other robotic-assisted bariatric procedures (26, 32, 33). In addition to which steps (or patient factors) influenced efficiency and variability, this study also identified objective metrics that quantify what surgeon behaviors within the most influential step—stomach dissection—differed most over the surgeon learning curve. Specifically, we used OPIs as objective measures, which were derived from three major surgical robotic system events: camera movement, energy activation, and arm swap. In addition to performing the multi-stage regression analysis across the entire case series, a second analysis was performed comparing earlier vs. later cases in the series to determine if there was any change over time. Counts of energy activation per minute was the top ranked OPI, which might be linked to dissection technique and surgeon technical skill using the energy pedals. By focusing training on related surgeon behaviors, one might allow for improved efficiency and reduced variability. Furthermore, the OPIs reported here removed the subjectivity inherent to rating scales (e.g., GEARS) and enabled scalability by eliminating the reliance on experts or crowds of lay people to complete the ratings.

This study has several limitations. First, this was a case series by a single surgeon across two institutions, and thus the identified dominant factors associated with efficiency and variability need to be reproduced by other surgeons and institutions to evaluate generalizability. Additionally, different surgical tasks and additional OPIs could be explored to see if they are more impactful to efficiency or variability. Community consensus across procedures will allow for more robust analysis and broad adoption (34). Finally, this work did not explore correlations between performance and additional, discrete outcomes, such as re-admission, re-operation, and blood transfusion. It will be important to focus future outcomes research in areas that could be significantly impacted by task-based surgeon performance vs. others that might be influenced by surgeon decisions (e.g., length of stay).

In future research, we plan to explore how these methods can be extended to account for variations in how surgery is delivered across institutions and geographies, and to examine other procedures and specialties and their main contributors to efficiency and variability. Additionally, we plan to incorporate more patient factors and outcomes to extend this work beyond efficiency. Related work has shown promising results that link OPIs from critical steps of robotic-assisted prostatectomy to outcomes (13, 14). Finally, we plan to develop machine learning techniques that overcome manual video annotation (11, 13, 35, 36).

## Conclusions

This study demonstrated the feasibility of using objective task analysis to identify main factors around surgeon and OR team behavior that influence overall procedure efficiency and variability. In particular, stomach dissection was identified as the most critical step, and energy activation rate within stomach dissection was the most critical behavior. Importantly, BMI did not influence overall efficiency of the surgeon, suggesting robotic-assisted surgery might decouple patient BMI and surgical efficiency. This is particularly important to deliver minimally invasive surgery to bariatric patients. We believe this data-driven objective task analysis approach could be used to provide actionable, surgeon-specific feedback that may also be generalized to be used by clinical teams to quantify and influence best practices for those aspects of surgery contributing most to overall efficiency and consistency.

## Data Availability Statement

The original contributions presented in the study are included in the article/Supplementary Material, further inquiries can be directed to the corresponding author.

## Ethics Statement

Ethical review and approval was not required for the study on human participants in accordance with the local legislation and institutional requirements. Written informed consent for participation was not required for this study in accordance with the national legislation and the institutional requirements.

## Author Contributions

MT performed the surgical procedures, collected data, contributed to study design, manuscript drafting, and revision. XL and ME performed statistical analysis, contributed to manuscript drafting, and revision. AJ contributed to study design, manuscript drafting, and revision. All authors read and approved the final manuscript.

## Conflict of Interest

MT, XL, ME, and AJ were employees of Intuitive Surgical, Inc. However, MT was not affiliated with nor funded by Intuitive Surgical when the procedures were performed.

## Publisher's Note

All claims expressed in this article are solely those of the authors and do not necessarily represent those of their affiliated organizations, or those of the publisher, the editors and the reviewers. Any product that may be evaluated in this article, or claim that may be made by its manufacturer, is not guaranteed or endorsed by the publisher.
