# Supplementary material for: Identification of Main Influencers of Surgical Efficiency and Variability Using Task-Level Objective Metrics: A Five-Year Robotic Sleeve Gastrectomy Case Series
Source: Front Surg. 2022 May 2;9:756522. doi: 10.3389/fsurg.2022.756522 (PMC9108208; doi:10.3389/fsurg.2022.756522)
Supplement: Supplementary file 1 [file Data_Sheet_1.PDF]

Supplementary Material

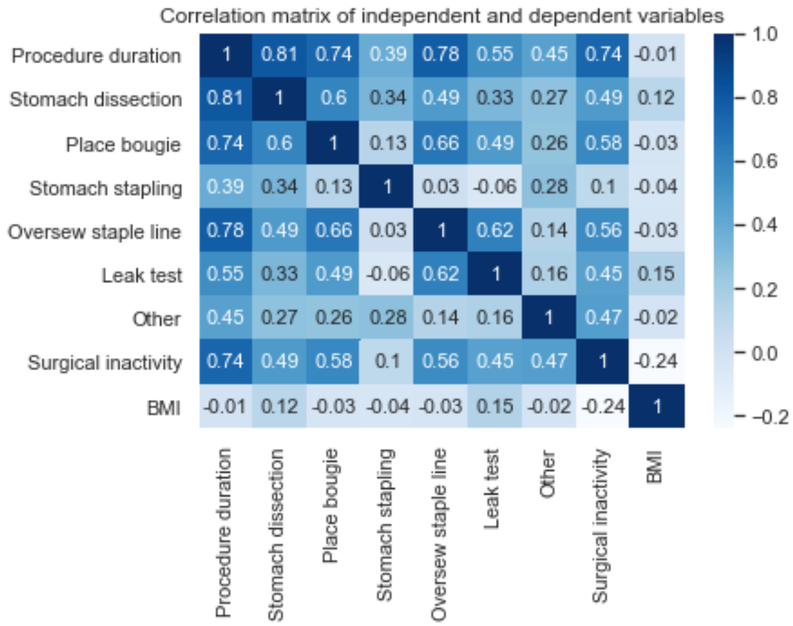

Supplementary Figure 1 Spearman correlation test between BMI, task durations and procedure duration. Stomach dissection duration has the highest correlation (R = 0.81) with procedure time. None of the independent variables were found to be highly correlated with each other (R ranging from –0.24 to 0.66).

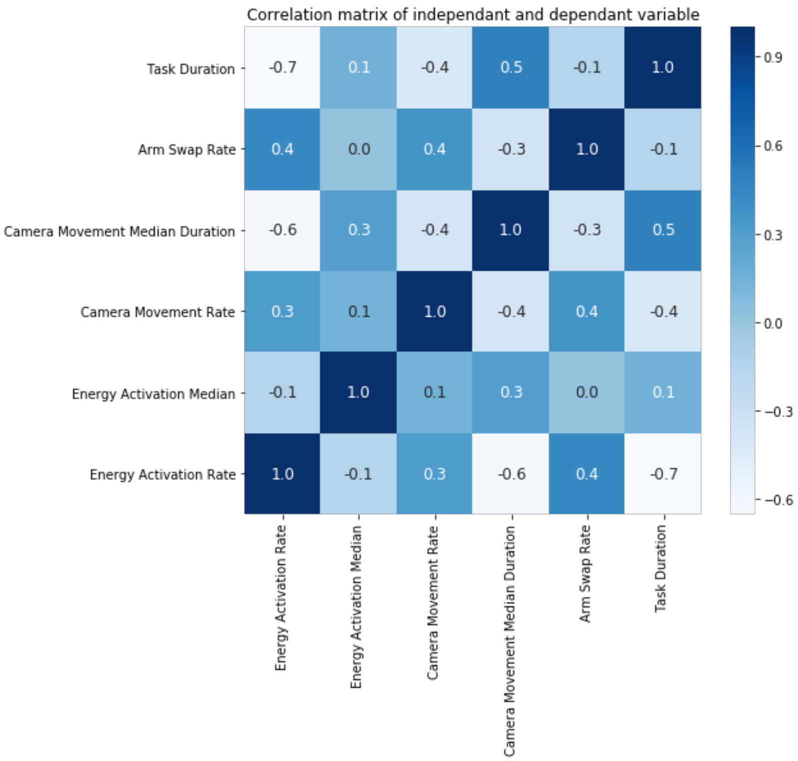

Supplementary Figure 2 Spearman correlation test between OPIs and stomach dissection duration. The absolute values of the correlation coefficients between each pair of OPIs were in the range of (0.01, 0.44) ensuring no multicollinearity.

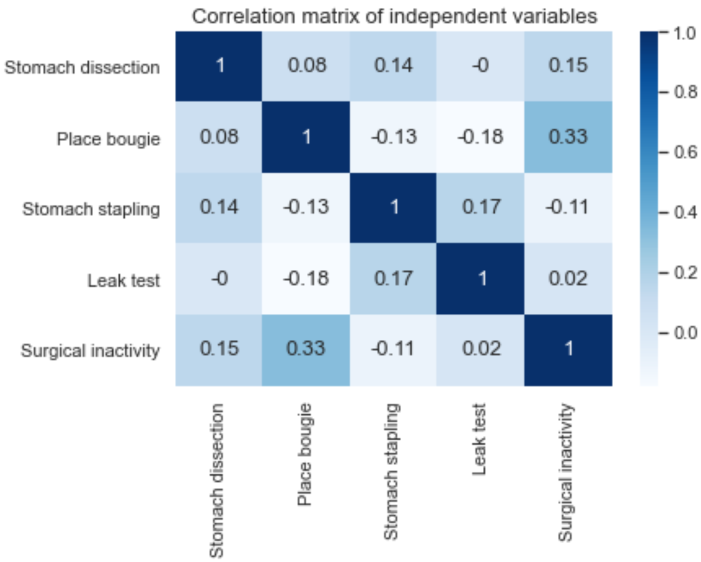

Supplementary Figure 3 Spearman correlation test of task duration IQRs for variability analysis. None of the independent variables were found to be highly correlated (coefficients ranging from – 0.18 to 0.33).
